# Triple-modality management of complex septated chronic subdural hematoma: a preliminary technical note on feasibility and safety

**DOI:** 10.3389/fneur.2026.1862361

**Published:** 2026-05-26

**Authors:** Fei Liu, Long Chen, Xueyi Wang, Liang Zhao, Zhiyang Li, Xiaojian Wang, Ping Song, Long Zhou, Zohaib Shafiq, Qiang Cai

**Affiliations:** 1 Department of Neurosurgery, The First Affiliated Hospital of Anhui Medical University, Hefei, China; 2 Department of Neurosurgery, Renmin Hospital of Wuhan University, Wuhan, China

**Keywords:** chronic subdural hematoma, dexamethasone, middle meningeal artery embolization, neuroendoscopy, technical note, triple-modality therapy

## Abstract

**Background:**

Complex septated chronic subdural hematomas (CSDHs) carry a high risk of recurrence following conventional drainage. This is hypothesized to be driven by three factors: neoangiogenesis, mechanical sequestration by fibrous septa, and a localized inflammatory microenvironment.

**Objective:**

To introduce and evaluate the preliminary safety and feasibility of a “Triple-Modality” surgical strategy addressing these components.

**Methods:**

We retrospectively reviewed 7 consecutive patients with complex, trabecular-type CSDHs who underwent a unified, single-session protocol: (1) Middle meningeal artery (MMA) embolization; (2) Neuroendoscopic fenestration; and (3) Intraoperative and postoperative subdural irrigation with a dexamethasone-saline solution (10 mg/500 mL). Outcomes were compared to an exploratory historical cohort of 11 similar patients treated with conventional burr-hole drainage.

**Results:**

The protocol was successfully applied to all 7 patients (median age 71 years). The median radiographic hematoma volume reduction was >95%. Over a median follow-up of 6.5 months, all patients improved to a Markwalder Grade of 0–1, with no recurrences (0/7, 0%). By contrast, the historical control cohort experienced a 36.3% (4/11) recurrence rate (*p* = 0.106). There were no steroid-related systemic complications, subdural empyemas, or wound healing defects observed in the experimental cohort.

**Conclusion:**

This preliminary technical note demonstrates that combining MMA embolization, endoscopic fenestration, and localized dexamethasone irrigation is technically feasible and safe. While early results suggest excellent radiographic clearance and low recurrence in highly complex CSDHs, larger prospective cohorts are necessary to evaluate the independent efficacy of each modality.

## Introduction

Chronic subdural hematoma (CSDH) is increasingly recognized not merely as a passive venous collection, but as an active inflammatory and neoangiogenic disorder (1, 2). This pathophysiology presents unique surgical challenges in “complex” CSDHs. Under the widely utilized Nakaguchi classification, the “trabecular type” is characterized by dense internal fibrous septations that mechanically compartmentalize the subdural space (3). These isolated loculations can act as active biochemical reservoirs, sequestering high concentrations of vascular endothelial growth factor (VEGF) and pro-inflammatory cytokines that continuously drive fluid exudation (4, 5). Consequently, complex CSDHs are frequently resistant to standard burr-hole decompression, yielding recurrence rates historically reported between 15 and 30% (3, 6).

Recent advancements have attempted to target these underlying mechanisms. Middle meningeal artery (MMA) embolization effectively reduces neoangiogenic blood supply (7–10), while neuroendoscopic evacuation allows for visually guided mechanical disruption of septal barriers (11–15). However, the local chemical driver—the intense inflammatory microenvironment of the neomembrane—often remains unaddressed by mechanical interventions alone. While systemic corticosteroids can blunt this inflammatory cascade, their use in elderly populations is limited by severe systemic side effects, including immunosuppression, hyperglycemia, delirium, gastrointestinal bleeding, and poor wound healing. Studies have demonstrated that while systemic steroids may reduce recurrence, this benefit is offset by significantly increased complication rates (16, 17).

To overcome this limitation, we developed a targeted intraoperative and postoperative dexamethasone irrigation protocol that delivers a potent corticosteroid directly to the active neomembrane surface, achieving supratherapeutic local concentrations while avoiding systemic toxicity. This technical note describes a combined “Triple-Modality Synergy” strategy integrating upfront MMA embolization to devascularize the surgical corridor, neuroendoscopic fenestration to mechanically unify the cavity, and this localized dexamethasone irrigation to neutralize inflammatory cytokines.

## Materials and methods

### Patient selection

Following Institutional Review Board approval (Approval no. PJ2021-03-31), we retrospectively reviewed 7 consecutive patients treated with this triple-modality approach between January 2025 and December 2025. Patient inclusion was strictly reserved for symptomatic CSDHs exhibiting “trabecular type” architecture on preoperative non-contrast CT, defined by the Nakaguchi classification as the presence of high-density septations separating the hematoma into multiple distinct compartments (3). MRI was utilized only if CT findings were equivocal. Patients with simple, homogenous CSDHs were excluded from this protocol to avoid excessive treatment.

### Triple-modality clinical algorithm

**Figure 1:**
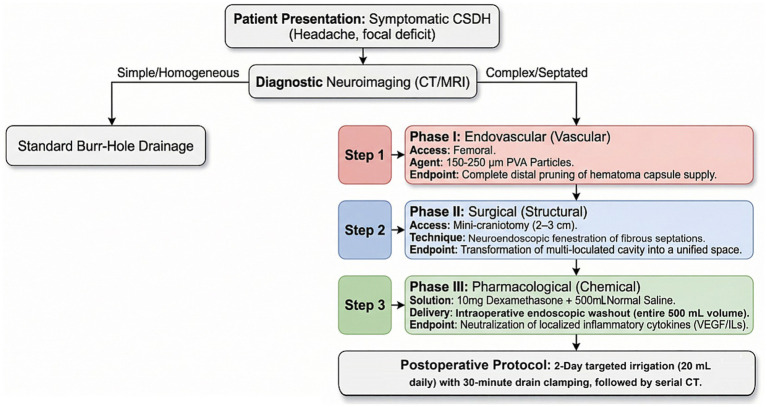
Clinical decision and operative algorithm for triple-modality management. This workflow illustrates the sequential, single-session protocol designed to systematically address the biological, structural, and chemical drivers of complex CSDH. Following patient selection via neuroimaging, the pathway integrates upfront MMA devascularization (Phase I), direct neuroendoscopic mechanical fenestration (Phase II), and targeted anti-inflammatory dexamethasone irrigation both intraoperatively and postoperatively (Phase III) to achieve comprehensive disease clearance and symmetrical brain re-expansion.

The procedure is executed as a sequential workflow (Figure 1).

### Phase 1: endovascular devascularization

**Figure 2:**
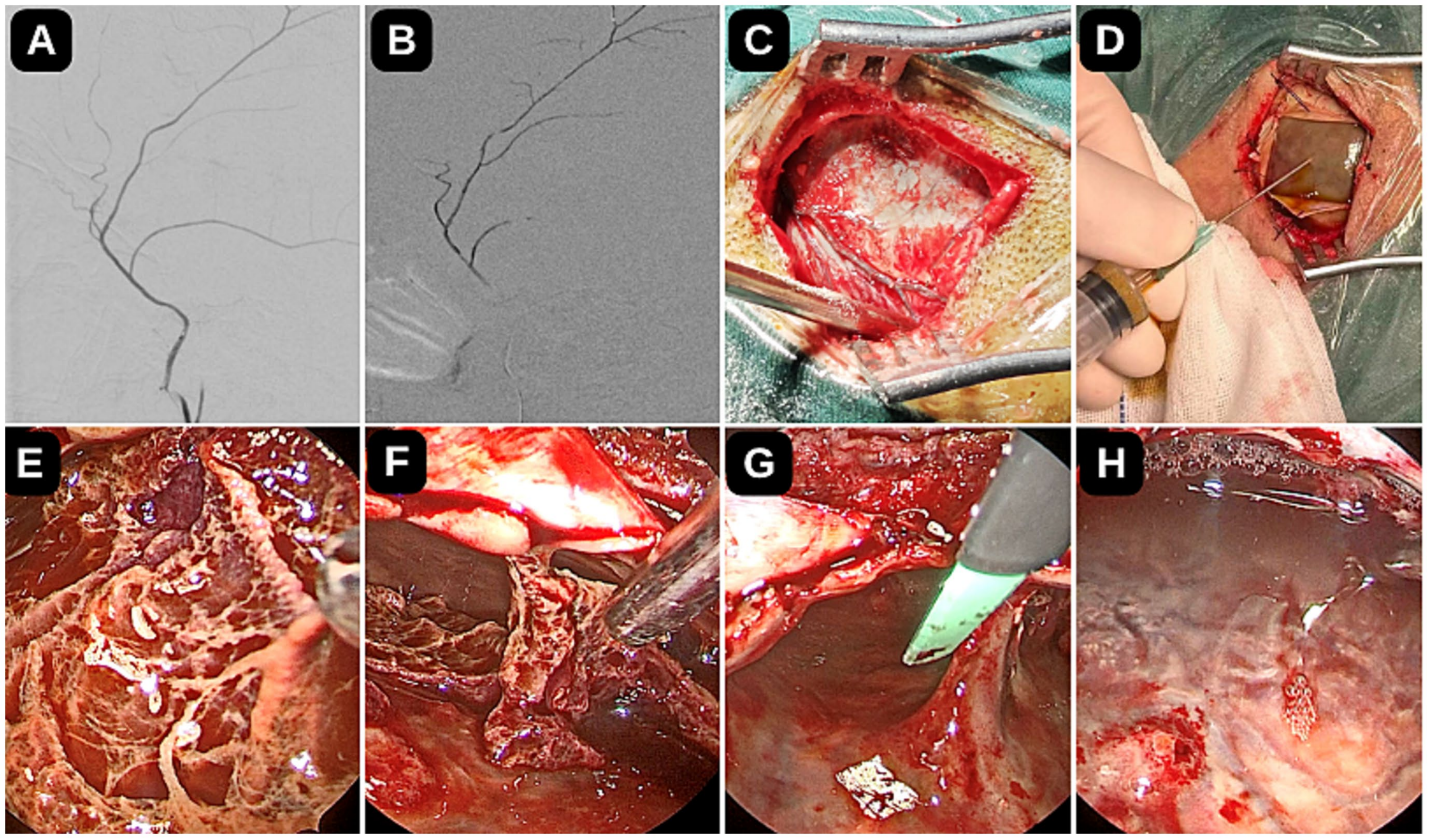
Stepwise execution of the triple-modality strategy in complex CSDH. An eight-panel intraoperative sequence illustrating the integration of endovascular, neuroendoscopic, and pharmacological interventions. **(A,B)** Phase 1: Endovascular devascularization. **(A)** Baseline lateral angiogram demonstrating the arterial supply to the hematoma capsule via the MMA. **(B)** Post-embolization angiogram confirming complete distal pruning and devascularization following the injection of PVA particles. **(C,D)** Phase 2 initialization: Surgical access. **(C)** Macroscopic exposure following mini-craniotomy and dural retraction, revealing the intact, avascular outer neomembrane. **(D)** Initial decompression via needle puncture and aspiration to safely reduce intracranial pressure prior to endoscope insertion. **(E–G)** Phase 2: Neuroendoscopic fenestration. **(E)** Initial endoscopic view revealing a highly complex, multi-loculated architecture with dense fibrous septations trapping organized clot. **(F)** Active mechanical fenestration and tearing of the internal trabeculae using endoscopic instruments within the optimized, bloodless corridor. **(G)** Complete unification of the subdural cavity, providing an unobstructed anatomical space. **(H)** Phase 3: Pharmacological stabilization. Continuous intraoperative irrigation of the unified space with a targeted dexamethasone-saline solution (10 mg/500 mL) to wash out organized clot debris and neutralize the inflammatory neomembrane surface.

Under general anesthesia, standard femoral arterial access is obtained. Targeted embolization of the MMA is performed using polyvinyl alcohol (PVA) particles (150–250 μm) under continuous fluoroscopic guidance. Particle agents are selected to achieve distal penetration without creating radiopaque beam-hardening artifacts on subsequent CTs (18). Embolization proceeds until angiographic pruning of the distal MMA branches supplying the hematoma capsule is confirmed (Figure 2).

### Management of Markwalder Grade 3 patients

**Figure 3:**
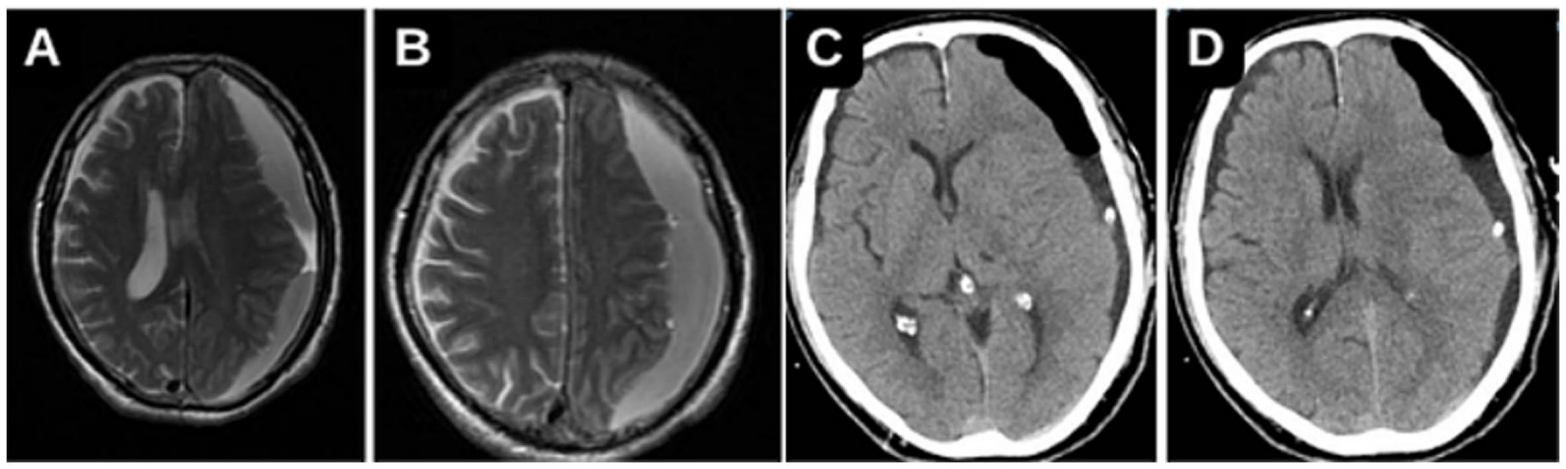
Radiological presentation and surgical outcome for case 1. **(A,B)** Preoperative imaging indicating significant layering and separation within the complex septated chronic subdural hematoma, causing associated mass effect. **(C,D)** The corresponding postoperative CT scan demonstrates 96% hematoma volume reduction and symmetrical brain re-expansion of the subdural collection.

Regarding patients with Markwalder Grade 3 (somnolent but responsive), the MMA embolization procedure adds approximately 30–45 min to the total operative time. In our cohort, all Grade 3 patients were clinically stable with no signs of impending herniation (defined as progressive anisocoria, worsening Glasgow Coma Scale, or brainstem reflex loss). The upfront embolization was performed under the same general anesthesia as the subsequent endoscopic evacuation, with continuous neuromonitoring. The delayed decompression is justified by the critical advantage of entering a devascularized surgical field, which dramatically reduces intraoperative “red-out” and allows for complete septal fenestration. For patients with Grade 4 (comatose) or signs of acute deterioration, we would bypass embolization and proceed directly to emergent surgical decompression—though no such patient was encountered in this series (Figure 3).

### Phase 2: Neuroendoscopic fenestration

Following devascularization, a mini-craniotomy or enlarged burr hole (2–3 cm) is created. A neuroendoscope is introduced into the subdural space. Utilizing endoscopic micro-forceps, the surgeon directly visualizes and fenestrates the internal septations and organized solid clots. Pre-emptive MMA embolization provides a relatively avascular corridor, mitigating the continuous capsular micro-bleeding that traditionally obscures the endoscopic lens.

### Phase 3: Anti-inflammatory dexamethasone irrigation

**Figure 4:**
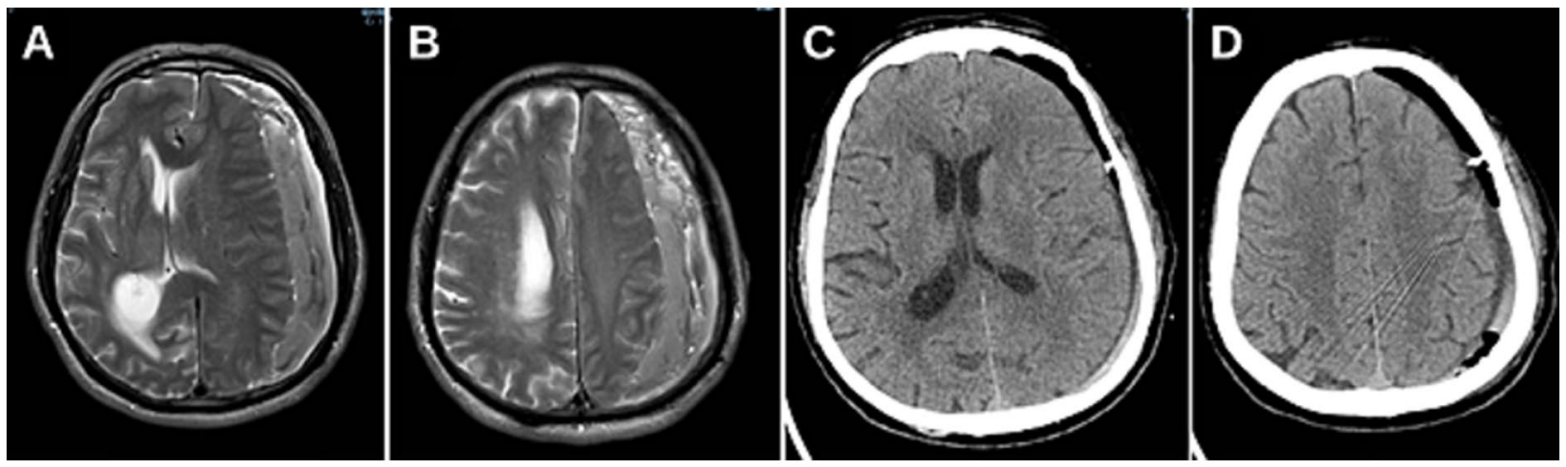
Radiological presentation and surgical outcome for case 2. **(A,B)** Preoperative imaging revealing a large, complex crescentic collection characterized by significant internal layering, separation, and mass effect with midline shift. **(C,D)** Postoperative CT scan shows 95% hematoma volume reduction, resolution of the compartmentalization, and restoration of normal intracranial anatomy following the triple-modality protocol.

Intraoperatively, the unified cavity is irrigated with 500 mL of body-temperature normal saline containing 10 mg of dexamethasone. A closed-system subdural drain is then placed under direct visualization. Postoperatively, for the first 2 days, 20 mL of a fresh dexamethasone-saline solution (0.4 mg dexamethasone equivalent) is instilled daily via the drainage tube. The drain is clamped for 30 min to allow therapeutic contact with the neomembrane before being released back to gravity drainage. The duration is strictly limited to 48 h to minimize the risk of retrograde infection (Figure 4).

### Statistical analysis

**Figure 5:**
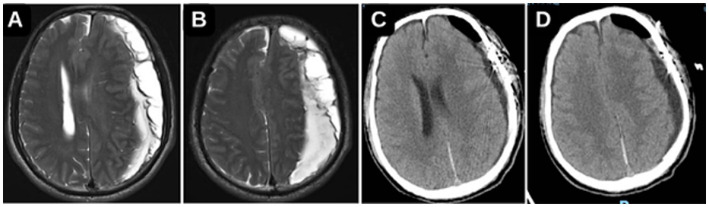
Radiological presentation and surgical outcome for case 3. **(A,B)** Preoperative imaging highlighting a large, highly loculated chronic subdural hematoma with a “honeycomb” appearance, demonstrating distinct multi-laminar stratification and separation. **(C,D)** The corresponding postoperative CT scan confirms 94% hematoma volume reduction and rapid brain re-expansion following the triple-modality intervention.

To contextualize our findings, clinical outcomes were compared against an exploratory historical cohort of 11 consecutive patients with complex trabecular CSDH treated with conventional burr-hole drainage at our institution in the preceding 12 months. Statistical analyses were performed using SPSS version 26.0. Continuous variables were compared using the Mann–Whitney U test, and categorical variables using Fisher’s Exact Test. A *p* < 0.05 was considered statistically significant (Figure 5).

## Results

The triple-modality approach was successfully applied to 7 patients (median age 71 years [range 52–76]). Baseline characteristics between the experimental cohort (*n =* 7) and the historical control cohort (*n =* 11) showed no significant differences in median age (*p* = 0.41) or preoperative Markwalder grade (*p* = 0.65).

Aggressive mechanical clearance via endoscopy resulted in a median radiographic hematoma volume reduction of >95%. Volume calculation method: Radiographic hematoma volume reduction was calculated using 3D Slicer software.^1^ Preoperative and postoperative CT scans were analyzed by two independent neuroradiologists blinded to clinical outcomes. On each axial slice, the hematoma region of interest was manually outlined, and total volume was calculated using the software’s segmentation module with pixel summation across all slices. Volume reduction percentage was defined as: [(Preoperative volume – Postoperative volume)/Preoperative volume] × 100%. The median reduction achieved was >95% (range 92–98%).

The median time to drain removal was 2 days, and median hospital stay was 5 days. Over a median follow-up of 6.5 months (range 6–12 months), all 7 patients improved to a Markwalder Grade of 0–1, with no clinical or radiographic recurrences (0%). In the historical cohort, 4 of 11 patients (36.3%) experienced a recurrence requiring reoperation within 6 months. While clinically notable, the difference in recurrence rates did not reach statistical significance due to the small sample size (*p* = 0.106).

### Safety profile

The targeted dexamethasone protocol was well-tolerated. No steroid-related systemic complications (e.g., clinically significant hyperglycemia >180 mg/dL) occurred. Importantly, despite the theoretical risk of local immunosuppression, there were no instances of delayed wound closure, incisional breakdown, subdural empyema, or meningitis at the 6-month follow-up (Tables 1, 2).

**Table 1:** Demographics and summary of outcomes for the triple-modality cohort (*N =* 7).

Patient	Age/Sex	Pre-op Markwalder grade	Post-op Markwalder grade (6-month)	Radiographic clearance	Recurrence/Complications
1	71/M	2	0	>96%	None
2	74/M	2	0	>95%	None
3	76/M	3	1	>94%	None
4	76/M	3	1	>95%	None
5*	52/M	1	0	>95%	None
6	71/M	2	0	>95%	None
7	63/M	2	0	>95%	None

*Patient 5 presented with a history of significant head trauma, predisposing him to CSDH at an earlier age.

**Table 2:** Standardized postoperative targeted dexamethasone irrigation protocol.

Postoperative phase	Clinical action	Volume and concentration	Clamping parameters	Primary rationale
Intraoperative (Day 0)	Continuous Active Washout	500 mL (10 mg Dexamethasone in 500 mL Normal Saline)	N/A (Continuous visualization)	Evacuate solid clot debris; initial widespread coating of unified neomembrane.
Postoperative (Days 1 and 2)	Targeted Bolus Instillation	20 mL (0.4 mg Dexamethasone equivalent)	Clamp drain for 30 min, then release to gravity.	Volume coats membrane without intracranial pressure spikes; time maximizes mucosal absorption half-life.
Postoperative (Day 3)	Drain Removal	N/A	N/A	Protocol limited to 48 h to capture acute inflammatory phase and minimize infection risk.

### Case illustrations

#### Case 1

A 71-year-old male presented with a 3-day history of paroxysmal headaches and mild right-sided hemiparesis. Neurological examination revealed mild generalized lethargy and slight right lower extremity weakness (preoperative Markwalder Grade 2). Non-contrast head CT confirmed a complex, trabecular CSDH with significant mass effect. The patient underwent our Triple-Modality protocol: initial selective MMA embolization achieved complete capsular devascularization, followed immediately by minimal-access neuroendoscopic fenestration, allowing for visually guided disruption of the internal trabeculae in a bloodless field. Finally, the cavity was irrigated intraoperatively with a 500 mL dexamethasone-saline solution. Postoperatively, the patient underwent the 48-h protocol consisting of a 20 mL daily irrigation volume followed by 30 min of drain clamping.

The patient tolerated the procedure without complications. The subdural drain was removed on postoperative day 2, and he was discharged on day 4. Postoperative imaging demonstrated excellent, symmetrical brain re-expansion with an estimated 96% reduction in hematoma volume. The patient achieved rapid clinical recovery (Markwalder Grade 0), with no recurrence noted at the 6-month follow-up.

#### Case 2

A 74-year-old male presented with spontaneous, progressive headaches, right-sided hemiparesis, and an unsteady gait (preoperative Markwalder Grade 2). A non-contrast head CT revealed a large, complex collection spanning the left convexity, characterized by indistinct borders, left ventricular compression, and rightward midline shift. Intraoperative neuroendoscopy successfully broke down the complex compartmentalization, allowing the dexamethasone irrigation to reach all subdural spaces. Following the standard 2-day postoperative clamping and irrigation regimen, the drain was safely removed on postoperative day 3. The patient’s hemiparesis resolved entirely (Markwalder Grade 0). Follow-up imaging confirmed optimal hematoma clearance (95% volume reduction) and complete resolution of the midline shift, with no recurrence at 6 months.

#### Case 3

A 76-year-old male presented with progressive cognitive decline and gait instability (preoperative Markwalder Grade 3). Preoperative imaging revealed a large, complex CSDH with thick internal septations, creating a highly loculated “honeycomb” appearance. Following MMA embolization to secure a bloodless field, neuroendoscopic intervention systematically disrupted the septations. Intraoperatively, the cavity was irrigated with 500 mL of the dexamethasone-saline solution, followed by the postoperative 20 mL irrigation bolus with 30-min clamping for two days. The patient was discharged on day 6. Follow-up CT imaging showed near-complete hematoma clearance (94% volume reduction) and symmetrical brain re-expansion. The patient’s neurological symptoms improved significantly (Markwalder Grade 1), and no recurrence was noted at the 6-month follow-up.

## Discussion

The surgical management of multiloculated, trabecular CSDH remains challenging due to the interplay of vascular neoangiogenesis, mechanical sequestration, and localized inflammation. Conventional monotherapies often address only one facet of this triad (6, 19).

This preliminary technical note explores the feasibility of bundling three targeted therapies. Upfront MMA embolization limits neoangiogenesis (10, 20–22) but relies on gradual resorption. Endoscopy provides immediate mechanical decompression (11–15) but can trigger a secondary inflammatory response due to mechanical trauma to the friable neomembrane. While recent reports have begun to highlight the feasibility of combining MMA embolization with endoscopic treatment to address both structural and vascular drivers (23, 24), the local chemical driver remains unaddressed. By introducing a localized intra- and postoperative dexamethasone irrigation, the inflammatory cascade can be blunted directly at the tissue level.

The application of topical corticosteroids (10 mg/500 mL) mirrors established protocols in other neurosurgical domains, such as peripheral nerve surgery and irrigation of tumor resection cavities. This targeted approach stabilizes the fragile capillary endothelium and downregulates localized VEGF production without triggering the severe systemic morbidities that limit oral corticosteroid use in the elderly (1, 5).

### Systemic steroid risks and rationale for local delivery

Systemic corticosteroids are associated with significant morbidity in the elderly CSDH population. A recent propensity-matched study by Thakur et al. (16) demonstrated that adjunctive systemic steroids did not improve outcomes following surgical evacuation with MMA embolization but were associated with increased rates of hyperglycemia and infectious complications. Similarly, a meta-analysis by Shrestha et al. (post-DEX-CSDH trial) confirmed that while oral steroids modestly reduce recurrence, this benefit is counterbalanced by a significantly elevated risk of adverse events including gastrointestinal bleeding, delirium, and poor wound healing (17). These data reinforce the approach of delivering dexamethasone directly into the subdural space—achieving high local concentrations at the neomembrane surface while virtually eliminating systemic exposure and its attendant risks.

The potential addition of oral steroids for sustained anti-inflammatory effects warrants consideration but is not supported by current evidence. The postoperative irrigation protocol used here (20 mL of dexamethasone-saline solution over 2 days with 30-min clamping) provides targeted local exposure during the critical 48-h acute inflammatory window after endoscopic septal disruption. Published pharmacokinetic data suggest that a single topical corticosteroid application achieves detectable anti-inflammatory effects lasting 24–72 h at mucosal surfaces, likely sufficient to down regulate the immediate cytokine surge triggered by mechanical fenestration. Extending therapy beyond 48 h would require prolonged drain retention, thereby increasing retrograde infection risk—a concern the present protocol was explicitly designed to minimize. Moreover, transitioning to oral steroids would reintroduce the systemic side-effect profile (hyperglycemia, immunosuppression, delirium) that the localized approach successfully avoids. Nevertheless, the optimal duration of local steroid exposure remains undefined. Future dose-escalation or comparative studies could evaluate whether an extended postoperative irrigation protocol (e.g., 4–5 days) or a single preoperative systemic dose provides additional benefit without excess morbidity.

The preliminary safety data support this localized approach, as no local infections, delayed healing, or systemic metabolic derangements were observed, likely due to the strict 48-h limitation of the postoperative clamping protocol.

### Limitations

Several critical limitations must be acknowledged. First, the sample size is extremely small (*n =* 7), and the comparison with a historical cohort introduces inherent selection biases; therefore, our statistical comparisons are strictly exploratory. Second, because three modalities were introduced simultaneously, it is currently impossible to statistically isolate the independent clinical contribution of the localized dexamethasone from the mechanical clearance achieved via endoscopy. We acknowledge that this combined approach is highly resource-intensive and represents an aggressive treatment strategy; hence, it should be strictly reserved for complex, trabecular-type CSDHs with a high inherent risk of recurrence, rather than homogenous collections.

## Conclusion

The integration of upfront MMA embolization, neuroendoscopic fenestration, and localized dexamethasone irrigation is a safe and technically feasible strategy for the management of highly complex, septated CSDHs. Preliminary results suggest excellent radiographic clearance and low recurrence rates without systemic steroid toxicity. However, larger, prospective, multicenter trials are necessary to validate these findings and elucidate the independent efficacy of each modality.

## Data Availability

The raw data supporting the conclusions of this article will be made available by the authors, without undue reservation.

## Footnotes

1
http://www.slicer.org
